# Monitoring Gaseous CO_2_ and Ethanol above Champagne Glasses: Flute versus Coupe, and the Role of Temperature

**DOI:** 10.1371/journal.pone.0030628

**Published:** 2012-02-08

**Authors:** Gérard Liger-Belair, Marielle Bourget, Hervé Pron, Guillaume Polidori, Clara Cilindre

**Affiliations:** 1 Equipe Effervescence, Groupe de Spectrométrie Moléculaire et Atmosphérique, UMR CNRS 7331, UFR Sciences Exactes et Naturelles, BP 1039, Reims, France; 2 Laboratoire d'Oenologie et Chimie Appliquée, Université de Reims, URVVC-SE UPRES EA 2069, BP 1039, Reims, France; 3 Laboratoire de Thermomécanique, Groupe de Recherche en Sciences pour l'Ingénieur, Université de Reims, BP 1039, Reims, France; 4 Laboratoire Stress, Défenses et Reproduction des Plantes, Université de Reims, URVVC-SE UPRES EA 2069, BP1039, Reims, France; German Cancer Research Center, Germany

## Abstract

In champagne tasting, gaseous CO_2_ and volatile organic compounds progressively invade the headspace above glasses, thus progressively modifying the chemical space perceived by the consumer. Simultaneous quantification of gaseous CO_2_ and ethanol was monitored through micro-gas chromatography (μGC), all along the first 15 minutes following pouring, depending on whether a volume of 100 mL of champagne was served into a flute or into a coupe. The concentration of gaseous CO_2_ was found to be significantly higher above the flute than above the coupe. Moreover, a recently developed gaseous CO_2_ visualization technique based on infrared imaging was performed, thus confirming this tendency. The influence of champagne temperature was also tested. As could have been expected, lowering the temperature of champagne was found to decrease ethanol vapor concentrations in the headspace of a glass. Nevertheless, and quite surprisingly, this temperature decrease had no impact on the level of gaseous CO_2_ found above the glass. Those results were discussed on the basis of a multiparameter model which describes fluxes of gaseous CO_2_ escaping the liquid phase into the form of bubbles.

## Introduction

Since the end of the 17^th^ century, champagne is a world-wide renowned French sparkling wine. From a strictly chemical point of view, Champagne wines are multicomponent hydroalcoholic systems supersaturated with carbon dioxide (CO_2_) dissolved gas molecules formed together with ethanol during the second fermentation process, called *prise de mousse* (promoted by adding yeasts and a certain amount of sugar inside bottles filled with a base wine and sealed with a cap). During the *prise de mousse*, bottles are sealed, so that CO_2_ molecules cannot escape and progressively dissolve into the wine [1]–[3]. Champagne wines therefore hold a concentration of dissolved CO_2_ proportional to the level of sugar added to promote this second fermentation. Actually, a standard 75 centiliters champagne bottle typically holds about 9 grams of dissolved CO_2_, which correspond to a volume close to 5 liters of gaseous CO_2_ under standard conditions for temperature and pressure [1]–[3].

When champagne is poured into a glass, there are indeed two pathways for progressive CO_2_ and volatile organic compounds (VOC) losses. CO_2_ and VOCs escape (i) into the form of heterogeneously nucleated bubbles, the so-called effervescence, and (ii) by “invisible” diffusion through the free surface of the glass [4]–[6]. Glass-shape, and especially its open aperture, is therefore also suspected to play an important role as concerns the kinetics of CO_2_ and flavor release during champagne tasting [5], [6]. From the consumer point of view, the role of bubbling is indeed essential in champagne, in sparkling wines, and even in any other carbonated beverage. Without bubbles, champagne would be unrecognizable, beers and sodas would be flat. However, the role of effervescence is suspected to go far beyond the solely aesthetical point of view. Recently, by use of ultrahigh resolution mass spectrometry, it was indeed demonstrated that ascending bubbles radiate a cloud of tiny champagne droplets overconcentrated with compounds known to be aromatic or the precursors of aromas [7]. Moreover, it was also recently demonstrated that the continuous flow of ascending bubbles strongly modifies the mixing and convection conditions of the liquid medium [5], [6]. In turn, the CO_2_ discharge by diffusion through the free air/champagne interface may be considerably accelerated, as well as the release of the numerous VOCs, which both strongly depend on the mixing flow conditions of the liquid medium [8].

Carbon dioxide is a potent irritant in the nasal cavity, as are many other organic compounds [9]–[11]. Carbonation, or the perception of dissolved CO_2_, involves a truly multimodal stimulus. In addition to the tactile stimulation of mechanoreceptors, CO_2_ acts on both trigeminal receptors [12]–[16] and gustatory receptors [17], [18]. Both of these chemically induced sensations involve the carbonic anhydrase enzyme, which can convert CO_2_ to carbonic acid. For the sense of taste, the stimulation with CO_2_ appears to involve the extracellular anhydrase enzyme and the transient receptor potential (TRP) mechanism of sour receptor cells [17]. This is consistent with the enhancement of sour taste by the presence of CO_2_ and the suppression of sweetness [19], [20]. Moreover, given the involvement of TRP mechanisms in both nociception and temperature sensing, interactions between carbonation and temperature might be expected. Actually, an enhancement of irritation, tactile sensations, cooling, and cold pain have all been observed with carbonation of solutions served at low temperature (as are indeed Champagne wines, sparkling wines, and carbonated beverages in general) [21]–[23]. For recent and global overviews of how dissolved CO_2_ may promote chemically induced sensations in the oral and nasal cavities, see the review by Brand [24], and the most recent edition of the book by Lawless and Heymann [25]. Among all the numerous VOCs found in wines, ethanol is obviously the one which is the most concentrated [26]. Ethanol is an effective gustatory, olfactory and trigeminal stimulus [27]. In recent studies, it has been shown that variation of wine ethanol content significantly contributes to the partitioning of odorants molecules in the wine headspace by modification of their solubility [28]–[30]. Furthermore, from the taster's point of view, the perception of wine flavors was also found to be influenced by glass shape [31], [32]. For all the aforementioned reasons, no wonder that a very strong coupling therefore finally exists in champagne and sparkling wines tasting, between dissolved CO_2_, the presence of rising bubbles, glass shape, CO_2_ discharge and VOCs release.

In case of champagne and other sparkling wine tasting, two quite emblematic types of drinking vessels have coexisted for decades: (i) the classical flute, namely a long-stemmed glass with a deep tapered bowl and a narrow aperture, and (ii) the classical coupe, namely a shallower glass with a much wider aperture. Their very different geometrical properties are believed to confer them completely different sensory profiles. Advantages and disadvantages of both glass shapes have indeed long been debated in popular wine magazines, nevertheless without bringing any analytical data corresponding to each type of drinking vessel. Very recently only, CO_2_ fluxes outgassing from a standard Champagne wine were measured, whether it was served in a flute or in a coupe [33].

In the present work, a micro-gas chromatography (μGC) technique coupled with a thermal conductivity detector (TCD) was used, in order to sample the chemical space above a glass poured with champagne, in real tasting conditions. Simultaneous quantification of gaseous CO_2_ and ethanol concentrations was monitored, all along the first 15 minutes following pouring, depending on whether champagne was served into a flute or into a coupe. Moreover, a recently developed visualization technique based on infrared imaging has been used, thus revealing the clouds of gaseous CO_2_ (completely invisible in the visible light spectrum) desorbing from the liquid phase during champagne tasting, whether champagne is poured into a flute or into a coupe.

## Materials and Methods

### Champagne samples

A standard commercial Champagne wine (with 12.5% v/v ethanol), elaborated with a blend of 100% Chardonnay base wines (vintage 2007, cooperative Nogent l'Abbesse, Marne, France), was used for this set of experiments. Since their elaboration, bottles were stored in a cool cellar, at 12°C. Concentration of CO_2_ molecules dissolved in Champagne samples (before pouring) was determined using carbonic anhydrase (labeled C2522 Carbonic Anhydrase Isozyme II from bovine erythrocytes, and provided from Sigma-Aldrich, US). This method is thoroughly detailed in two recent papers [33], [34]. Before pouring, champagne was found to hold a concentration of dissolved CO_2_ of 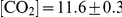 g/L.

Some classical physicochemical parameters of champagne samples were already determined at 20°C, with samples of champagne first degassed [4]. Its static surface tension, *γ*, was found to be of order of 50 mN m^−1^, and its density *ρ* was found to be very close to that of water, i.e., 10^3^ kg m^−3^. In the range of usual champagne tasting temperature (varying from approximately 5°C to 15°C), both surface tension and density of champagne are known to be very slowly temperature-dependent, contrary to its viscosity which is known to be strongly temperature-dependent. The temperature dependence of champagne, measured with a thermostated Ubelhode capillary viscosimeter (with a sample of champagne first degassed), was found to classically obey the following Arrhenius-like equation [4]:
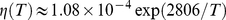
(1)where the dynamic viscosity *η* is expressed in mPa s, and the temperature *T* is expressed in K.

### Glass washing protocol

In order to avoid the randomly located “bubbling environment” inevitably provided in glasses showing natural effervescence [35], we decided to use, for this set of experiments, single standards flute and coupe with bubble nucleation sites artificially etched just above the central stem (thus providing a “standardized” and artificial effervescence). More details on artificial bubble nucleation provided by laser beam impacts can be found in ref [5]. Between the successive pouring and time series data recordings, the flute and the coupe were systematically thoroughly washed in a dilute aqueous formic acid solution, rinsed using distilled water, and then compressed air dried. This drastic treatment forbids the formation of calcium carbonate crystals on the flute wall as well as the adsorption of any dust particle acting as “natural” bubble nucleation sites. Therefore, with such a surface treatment, the CO_2_ bubble nucleation is strictly restricted to the bubble nucleation sites of the ring-shaped etching, so that differences in the kinetics of CO_2_ release from one type of drinking vessel to another are attributed only to geometrical differences between them.

### Micro gas chromatography procedure

μGC is generally employed to monitor gas of environmental interest such as CO_2_, N_2_O, CH_4_ [36], [37]. GC coupled with a thermal conductivity detector has already been applied to the analysis of CO_2_, N_2_ and O_2_ in beverage headspace, the respective concentration of each gas present being determined with a headspace sampler developed to puncture the beverage package (carbonated beverages or still wines) [38]. In the present study, analyses were conducted on a dual channel (A and B) micro gas chromatograph equipped with thermal conductivity detectors (TCD) (MicroGC 200, Agilent, SRA Instruments, France). On channel A, a PoraPlot U (PPU) column was set at 140°C for determination of CO_2_ while analysis of ethanol was performed on channel B with a OV-1 column at 100°C. Helium was used as a carrier gas in the two columns. The injection time on both columns was 50 ms. Chromatograms were obtained every 60 s. Peaks areas were quantified using the SOPRANE software (version 2.6.5). The chromatographic conditions for the analysis of CO_2_ and ethanol and the peak integration parameters used were the same as those previously described (**Table S1**) [39]. The quantity of CO_2_ was determined by means of a calibration curve using two bottles containing respectively 10% and 1% of standard CO_2_ (supplied by Linde gas, France) and air (≈0.038% of CO_2_) was used as a control. Ethanol was quantified with a bottle of gas holding 0.25% of standard ethanol (supplied by Linde gas, France). Calibration with the standard bottles were made with direct connection of the bottle to the μGC sample injection valve using stainless steel tubing, avoiding any gas loss or any disturbing airstream, thus keeping constant the concentration of standard gas. The gas delivery pressure was also kept constant to 1 bar. Then, analyses and quantification of standard gases for calibration were made with the same parameters as those used for the samples (**Table S1**). Calibration curves plotted the relative area of CO_2_ or ethanol versus the concentration of the standard. The procedure finally developed has shown a high reproducibility: 0.18% relative standard deviation (RSD) for CO_2_ 10%, 0.54% RSD for CO_2_ 1% and 0.28% RSD for ethanol.

### Experimental set-up and procedure used to measure the concentration of CO_2_ and ethanol above a glass poured with champagne

**Figure 1:**
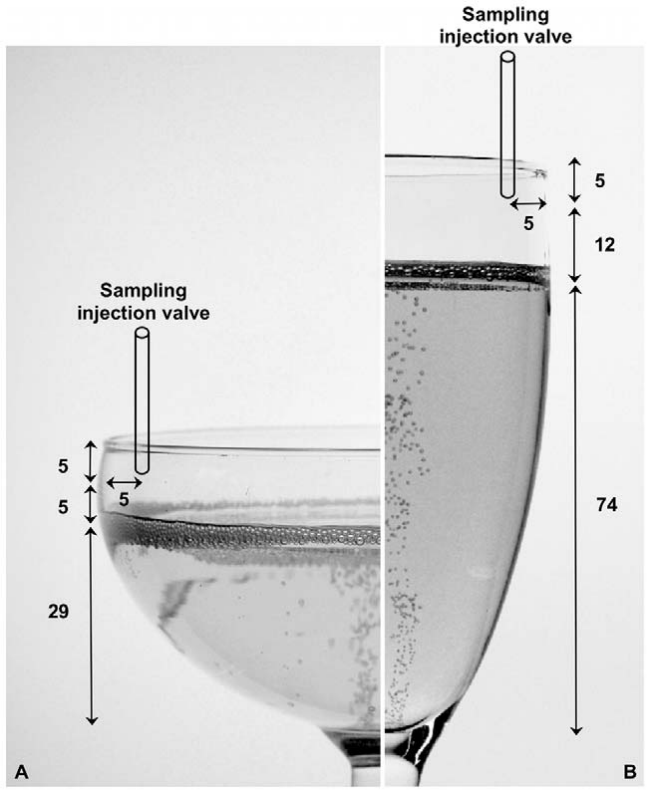
Headspace sampling injection valve positions above champagne glasses. Scheme illustrating the two well-defined valve sampling injection valve positions in the headspace above the champagne surface, whether champagne is served into the coupe (a) or into the flute (b) (dimensions are indicated in mm).

**Figure 2:**
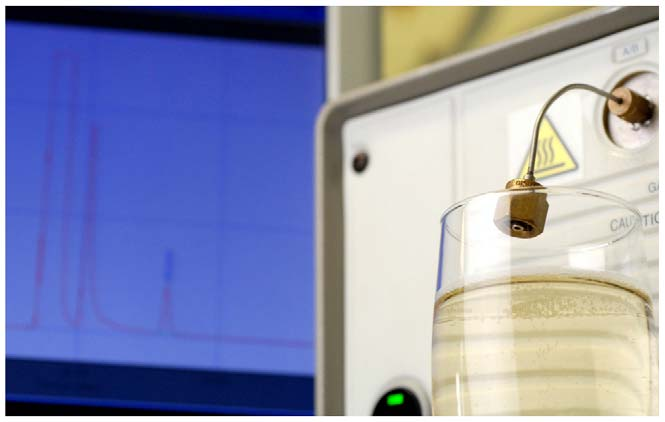
Gas chromatograph injection valve sampling gases in the headspace above the flute. Photograph by Gérard Liger-Belair.

A volume of 100±2 mL of champagne was carefully poured into the glass previously level-marked. During the standard champagne-like way of serving, champagne vertically falls and hits the bottom of the glass. Characteristic geometrical dimensions and liquid levels of the flute and coupe filled with 100 mL of champagne are displayed in **Figure 1**. Immediately after pouring, the glass was manually placed at a well-defined position under the injection valve of the chromatograph (see the scheme displayed in **Figure 1**). Then, the chromatographic analysis was started, and the sampling of champagne headspace above the glass was performed during 10 s and was repeated every 60 s, during 15 min following the pouring process. **Figure 2** presents a photographic detail of our experimental set-up. It is worth nothing that, in usual tasting conditions, the consumer rather “sniffs” at the edge of the glass. Therefore, we have decided to analyze CO_2_ and ethanol concentrations above the champagne surface, close to the edge of the glass for the following experiments.

Experiments were conducted at room temperature (23±2°C). Champagne wines were stored at 12 or 20±1°C for one day before the experiment. Between the successive pourings, bottles were hermetically closed and stored at the appropriate temperature. To enable a statistical treatment, six successive pouring and successive data recordings on three distinct bottles were done for both glass types.

Statistical analysis was done by Student's t test (two-tailed, two sample unequal variance) to determine whether concentrations of CO_2_ or ethanol were significantly different for each time after pouring. Differences at P<0.05 were considered as significant.

### Infrared imaging technique used to visualize the flow of gaseous CO_2_ desorbing from champagne

A visualization technique based on the InfraRed (IR) thermography principle has been used to film the gaseous CO_2_ fluxes outgassing from champagne (invisible in the visible light spectrum) [40]. The CO_2_ absorptions observable by the IR camera are quite weak because this gas molecule has only a strong absorption peak in the detector bandwidth at 4.245 µm. Consequently, the best way to visualize the flow of gaseous CO_2_ desorbing from champagne is to fit the IR video camera with a band-pass filter (centered on the CO_2_ emission peak). The experimental device consists of a *CEDIP middlewaves Titanium HD560M* IR video camera, coupled with a CO_2_ filter (Ø 50.8 mm×1 mm thick – *Laser Components SAS*). In complement, the technique involves an extended high-emissivity (0.97) blackbody (*CI systems* provided by *POLYTEC PI*), used at a controlled uniform temperature of 80°C, and placed approximately 30 cm behind the glass. The IR video camera was used at a 10 frames per second (fps) filming rate.

## Results and Discussion

### Losses of dissolved CO_2_ during the service of champagne in each type of drinking vessel

**Figure 3:**
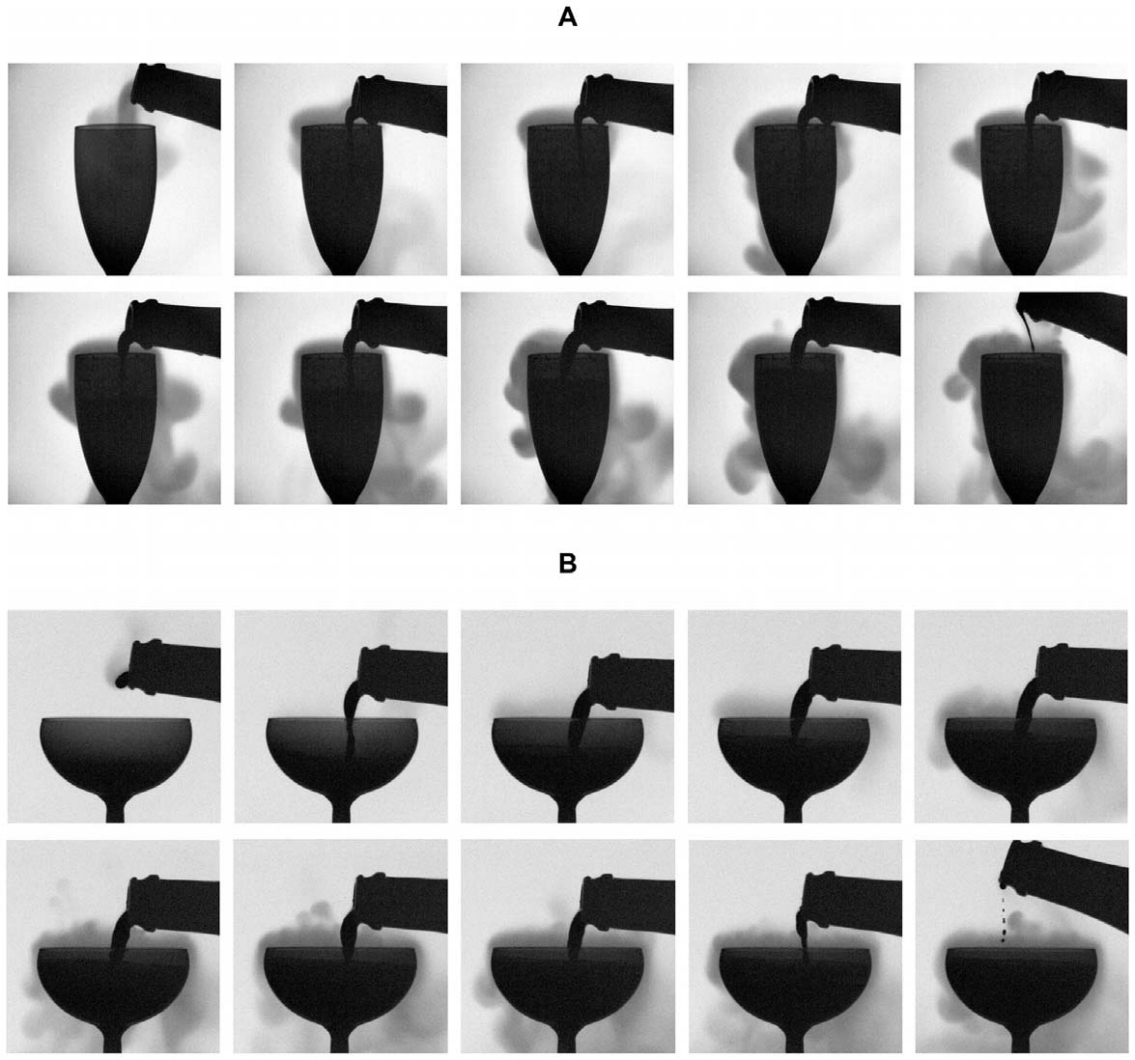
Infrared imaging of gaseous CO_2_ desorbing when pouring champagne into both glass types. Gray scale time-sequences illustrating the pouring step as seen through the objective of the IR video camera – for a bottle stored at 20°C – whether champagne is served into the flute (a) or into the coupe (b).

As recently shown in a previous article, the pouring process is far from being inconsequential with regard to the concentration of CO_2_ dissolved into the wine [41]. During the several seconds of the pouring process, champagne undergoes highly turbulent and swirling flows. During this phase, champagne loses a very significant part of its initial content in dissolved CO_2_. Gray scale infrared thermography time-sequences displayed in **Figure 3** illustrate the progressive losses of dissolved CO_2_ desorbing from the liquid phase into the form of a cloud of gaseous CO_2_, whether champagne is poured in a flute or in a coupe. Clouds of gaseous CO_2_ escaping from the liquid phase clearly appear. Consequently, at the beginning of the time series (i.e., at *t* = 0, after the glass was poured with champagne and manually placed below the sampling valve of the chromatograph), champagne holds a level of dissolved CO_2_ well below 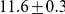 g L^−1^ (as chemically measured inside a bottle, after uncorking, but before pouring). In the present work, the initial bulk concentration of dissolved CO_2_ after pouring, denoted 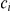, was also chemically accessed by using carbonic anhydrase. To enable a statistical treatment, six successive CO_2_-dissolved measurements were systematically done for each type of drinking vessel, after six successive pouring (from six distinct bottles). When served at 20°C, champagne was found to initially hold (at *t* = 0, after pouring) a concentration of CO_2_-dissolved molecules of 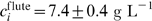 g L^−1^ in the flute, and 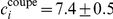 g L^−1^ in the coupe (i.e., approximately 4 g L^−1^ less in both types of drinking vessel after pouring than inside the bottle, before pouring).

### Gaseous CO_2_ and ethanol content found in the headspace above each type of drinking vessel

**Figure 4:**
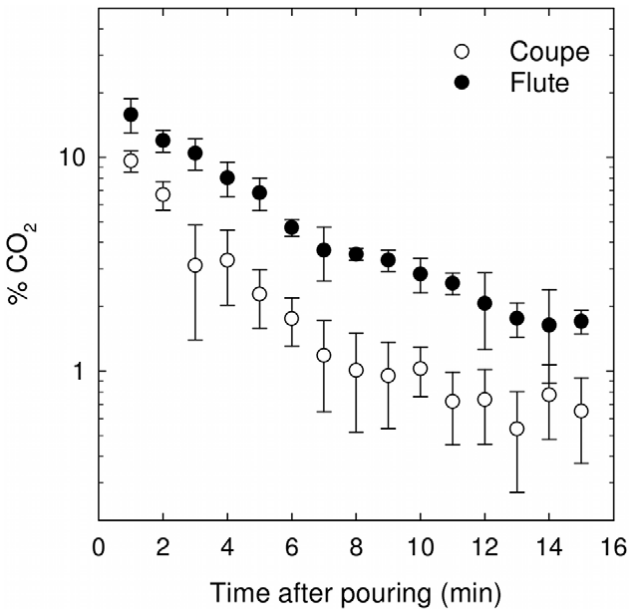
Monitoring gaseous CO_2_ concentrations in the headspace of a flute or a coupe filled with champagne. CO_2_ concentrations found in the headspace, all along the first 15 min after pouring champagne (for a bottle stored at 20°C), depending on whether champagne is served into the flute or into the coupe; each datum of each time series is the arithmetic average of six successive values recorded from six successive pouring; standard deviations correspond to the root-mean-square deviations of the values provided by the six successive data recordings.

**Figure 5:**
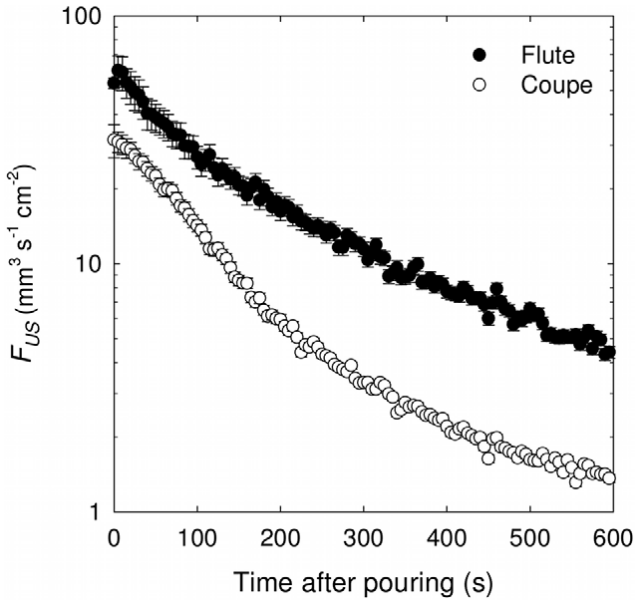
CO_2_ volume fluxes desorbing from a flute and a coupe. CO_2_ volume fluxes per unit surface (in mm^3^ s^−1^ cm^−2^) desorbing from a flute and a coupe, respectively filled with 100 mL of champagne (for a bottle stored at 20°C), all along the first 10 min following the pouring process (redrawn from Liger-Belair et al. [33]).

Concentrations of gaseous CO_2_ found above the wine surface were monitored during the first 15 minutes following pouring, as displayed in **Figure 4**, whether champagne was served into the flute or into the coupe. All along the first 15 minutes following pouring, concentrations of gaseous CO_2_ found close to the edge of the flute are approximately between two and three times higher than those reached above the coupe. This observation is self-consistent with some recent data about volume fluxes of gaseous CO_2_ measurements above glasses poured with champagne, including a flute and a coupe (as seen in the graph displayed in **Figure 5**) [41].

Fluxes of gaseous CO_2_ per unit surface area offered to gas discharging are indeed significantly higher above the surface of the flute than above the surface of the coupe because the same total amount of dissolved CO_2_ (≈0.7 gram for both glass types after pouring) has to be released by bubbles from a narrower surface, thus concentrating in turn more gaseous CO_2_ in the headspace above the flute. The observation reported in **Figure 4** is the analytical proof of a situation well-known by champagne and sparkling wines tasters. Actually, due to higher concentrations of gaseous CO_2_ above the flute than above the coupe, the smell of champagne, and especially its first nose, is always more irritating when champagne is served into a flute. It is indeed well-known that a sudden and abundant quantity of CO_2_ (a strong trigeminal stimulus) may irritate the nose during the evaluation of aromas [3]. Moreover, it clearly appears from **Figure 4** that the concentration of gaseous CO_2_ in the headspace above glasses progressively and quickly decreases as time proceeds. This observation betrays the fact that the dissolved CO_2_ content in the liquid phase also quickly decreases as time proceeds - from 7.4 g L^−1^ after pouring to about 3 g L^−1^ 15 minutes later in the case of the flute, as shown in a previous article [41] - thus decreasing in turn the rate at which gaseous CO_2_ escapes from the champagne surface.

In a previous article, a model was proposed, which links the total flux of gaseous CO_2_ molecules desorbing from the liquid phase into the form of bubbles (denoted 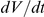) with several parameters of the liquid medium [34]:
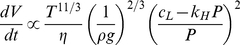
(2)with 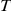 being the liquid temperature, *η* being the champagne viscosity, *ρ* being the champagne density, *g* being the acceleration due to gravity, 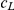 being the bulk concentration of dissolved CO_2_, 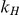 being the solubility of CO_2_ molecules in champagne, and *P* being the ambient pressure.

It is worth noting from eq (2) that the lower the dissolved CO_2_ concentration is in champagne, the lower the flux of gaseous CO_2_ desorbing from champagne into the form of collapsing bubbles. This is totally self-consistent with our μGC data displayed in **Figure 4** showing a progressive decrease of gaseous CO_2_ concentrations in the headspace above glasses (because the concentration of dissolved CO_2_ progressively decreases in champagne all along the first 15 minutes following pouring).

**Figure 6:**
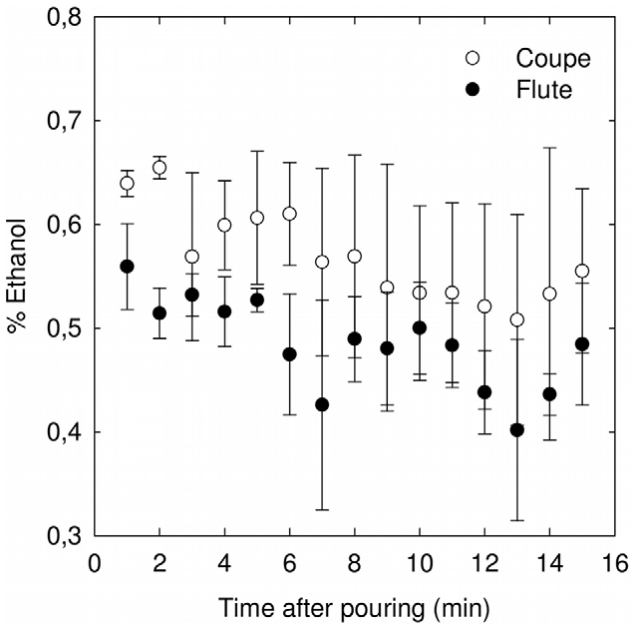
Monitoring ethanol concentrations in the headspace of a flute or a coupe filled with champagne. Ethanol concentrations found in the headspace above the champagne surface, all along the first 15 min after pouring champagne (for a bottle stored at 20°C), depending on whether champagne is served into the flute or into the coupe; each datum of each time series is the arithmetic average of six successive values recorded from six successive pouring; standard deviations correspond to the root-mean-square deviations of the values provided by the six successive data recordings.

Simultaneously, the concentration of ethanol was monitored with the same successive samplings of the champagne gaseous headspace, analyzed with the second module of the μGC. The successive levels of ethanol found in the headspace above both glass types, all along the first 15 min after pouring, are displayed in **Figure 6**. Quite surprisingly at first sight, and whatever the glass type, it is worth noting from **Figures 4** and **6** that the concentration of ethanol vapors remains roughly constant all along the 15 min following pouring, whereas the concentration of gaseous CO_2_ progressively and quickly decreases. The case of ethanol is indeed basically different. Actually, from a strictly chemical point of view, champagne is a highly complex water/ethanol mixture (at 12.5° v/v). Ethanol being more volatile than water, it evaporates more rapidly than water does. Nevertheless, during the first 15 minutes following pouring, the concentration of ethanol in the liquid phase remains roughly constant, because the “reservoir” of ethanol is huge (≈10 grams per glass) compared to the small reservoir of dissolved CO_2_ (≈0.7 gram per glass after pouring) which quickly decreases as time proceeds. Therefore, the rate at which ethanol vapors escape from the champagne bulk remains roughly constant (thus keeping roughly constant the concentration of ethanol vapors in the headspace).

**Figure 7:**
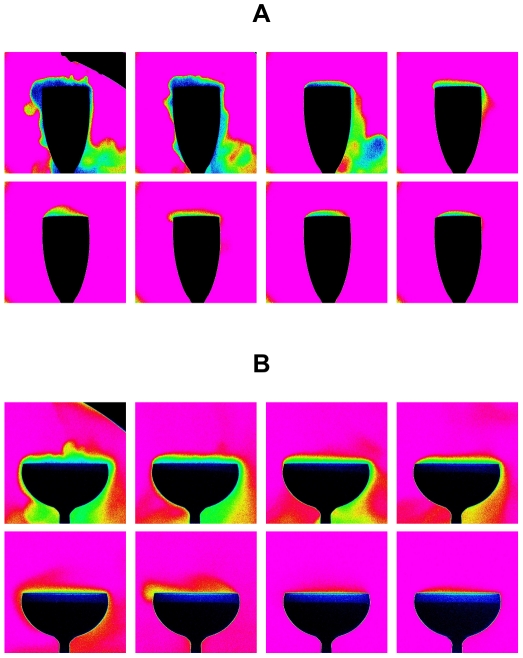
Infrared imaging of gaseous CO_2_ desorbing from glasses filled with champagne. False color time-sequences illustrating champagne glasses as seen through the objective of the IR video camera, after the pouring step – for a bottle stored at 20°C – whether champagne is served into the flute (a) or into the coupe (b). Zones highly concentrated in gaseous CO_2_ appear in black and dark blue, whereas zones slowly concentrated in gaseous CO_2_ appear in red.

**Figure 8:**
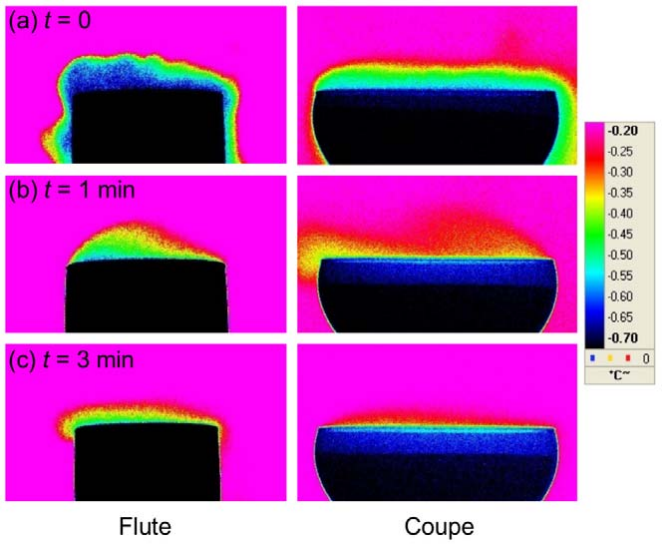
Close-up on gaseous CO_2_ desorbing above both glass types. False color IR time-sequences showing close-up snapshots of CO_2_ clouds desorbing above the flute and the coupe, respectively, immediately after pouring (a), 1 min after pouring (b), and 3 minutes after pouring (c); By using the color scale which provides a correspondence between the relative abundance of gaseous CO_2_ and the temperature detected by the IR sensor of the camera after absorption by the gaseous headspace above glasses, it clearly appears that gaseous CO_2_ is always more concentrated above the flute than above the coupe.

By using time-sequences provided through infrared imaging, the gaseous CO_2_ desorbing from champagne and progressively invading the headspace above glasses was made visible in a false color scale (see **Figure 7**). Such an image processing analysis provides a better visualization of the relative differences in the CO_2_ concentration field between both glass types, as shown in the thermography images displayed in **Figure 8**. Zones highly concentrated in gaseous CO_2_ appear in black and dark blue, whereas zones slightly concentrated in gaseous CO_2_ appear in red. The concentration of CO_2_ found above the flute (close to the edge) is indeed always significantly higher than that found above the coupe, thus confirming the tendency underscored through the μGC measurements. It can be noted for example, through infrared imaging, that the headspace (above the champagne surface, but below the glass edge) remains black during the first 3 min following pouring in case of the flute, whereas it progressively turns blue in case of the coupe. Moreover, it is also worth noting from infrared imaging time-sequences that the cloud of gaseous CO_2_ escaping from champagne tends to stagnate above the glass, or even tends to flow down from the edge of glasses by “licking” the glass walls (rather than diffuse isotropically around them). These observations conducted through infrared imaging betray the fact that gaseous CO_2_ is approximately 1.5 times denser (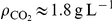 at 20°C) than dry air is (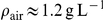 at 20°C), and therefore tends to naturally flow down.

### The impact of champagne temperature

**Figure 9:**
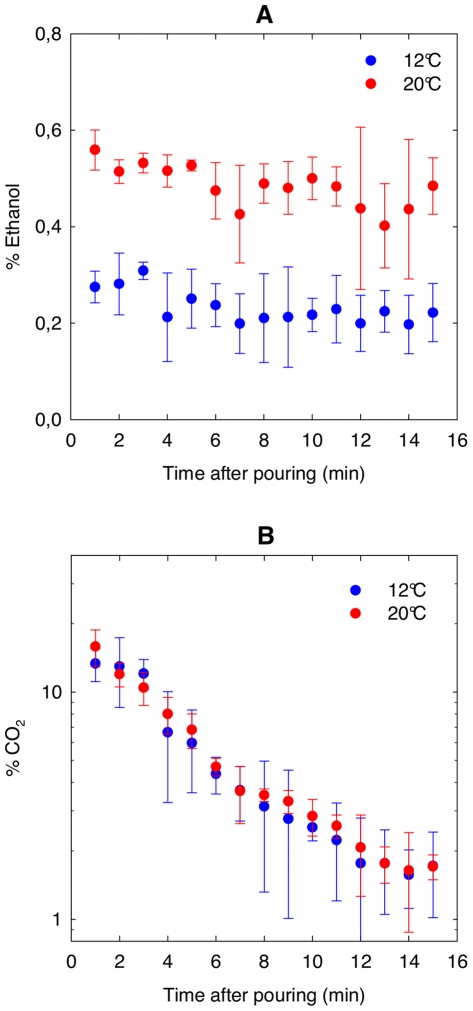
Monitoring ethanol and CO_2_ above a flute poured with champagne served at 20 or 12°C. Ethanol (A) and CO_2_ (B) concentrations found in the headspace above the champagne surface, all along the first 15 min after pouring champagne, depending on whether champagne is served at 20°C or at 12°C; each datum of each time series is the arithmetic average of six successive values recorded from six successive pouring; standard deviations correspond to the root-mean-square deviations of the values provided by the six successive data recordings.

The impact of champagne temperature on the progressive release of gaseous CO_2_ and ethanol desorbing from a flute was also investigated, through micro gas-chromatography. Champagne was served at 12°C, and the concentrations of gaseous CO_2_ and ethanol found in the headspace above champagne surface was compared with μGC data provided by the same champagne served at 20°C (**Figure 9**). As could have been expected, the headspace above the champagne surface is significantly less concentrated in vapors of ethanol when champagne is served at 12°C. Actually, the saturated vapor pressure of a liquid phase is indeed strongly temperature-dependent.

Generally speaking, the lower the temperature is, the lower the saturated vapor pressure of a liquid phase, and therefore the lower the rate of evaporation of the liquid phase. It is clearly self-consistent with the lower concentration of gaseous ethanol found at 12°C above the champagne surface rather than at 20°C, as seen in **Figure 9a**.

Nevertheless, and quite surprisingly, the case of gaseous CO_2_ seems rather different than that of ethanol, as can be seen in **Figure 9b**. Concentrations of gaseous CO_2_ found above the flute are of the same order of magnitude, and show the same general trend along the first 15 minutes following pouring, whether champagne is served at 12°C or at 20°C. The desorption of CO_2_ molecules from the liquid phase is fundamentally different than the desorption of ethanol. Ethanol progressively and continuously evaporates from champagne (as water does also) simply because the opened atmosphere above the flute never reaches the saturated vapor pressure of the binary water/ethanol mixture, whereas CO_2_ progressively diffuse from the liquid phase because champagne is supersaturated with dissolved CO_2_ molecules.

By replacing in eq (2) the viscosity by its Arrhenius-like expression given in eq (1), eq (2) transforms as:
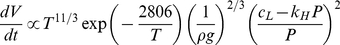
(3)The higher the champagne temperature is, the higher the flux of gaseous CO_2_ desorbing from the liquid phase into the form of bubbles. Theoretically, and following eq (3), the flux of gaseous CO_2_ desorbing from champagne into the form of bubbles should be about 40% higher at 20°C than at 12°C, every other parameter being equal under the same operating conditions. Nevertheless, and despite this strong dependence of CO_2_ fluxes on the champagne temperature, the concentration of gaseous CO_2_ above the champagne surface experimentally determined through the μGC procedure shows the same general trend and order of magnitude whether champagne is served at 12°C or at 20°C. We are surprised and logically tempted to wonder why.

An attempt to explain this experimental observation invokes both the role of the dissolved CO_2_ concentration 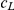 found in champagne, and the temperature dependence of the gaseous CO_2_ density. Actually, as shown in eq (3), the flux of gaseous CO_2_ desorbing from champagne depends on several parameters, including 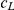 in the last term. Now, it is worth noting that losses of dissolved CO_2_ during the pouring of champagne into the flute strongly depend on the champagne temperature itself [41], [42]. Immediately after pouring and during the first 10 min following, champagne holds almost 1 g L^−1^ more dissolved CO_2_ when it is served at 12°C than when it is served at 20°C [41], [42]. Thus, at lower champagne temperatures, the last term of eq (3) should increase, thus counterbalancing the 40% decrease of the two first terms. Actually, by reducing the champagne temperature from 20°C to 12°C, the last term of eq (3) (which includes the “dissolved CO_2_” effect) experiences a rough 20% increase. The other 20% of gaseous CO_2_ needed to theoretically counterbalance the 40% decrease in the flux of gaseous CO_2_ desorbing from the champagne surface at 12°C could originate from a “density” effect. Actually, because the density of gaseous CO_2_ increases at lower temperature, the flux of gaseous CO_2_ desorbing from champagne at 12°C tends to naturally stagnate even more easily above the champagne surface than at 20°C. It could therefore lead to higher gaseous CO_2_ concentrations above the champagne surface than theoretically expected from fluxes of gaseous CO_2_ as described by eq (3).

In conclusions, simultaneous monitoring of gaseous CO_2_ and ethanol was conducted, through micro-gas chromatography (μGC), all along the first 15 minutes following pouring, depending on whether a volume of 100 mL of champagne was served into a flute or into a coupe. The concentration of gaseous CO_2_ was found to be significantly higher above the flute than above the coupe. Moreover, a recently developed gaseous CO_2_ visualization technique based on infrared imaging was performed, thus confirming this tendency. Those analytical results are self-consistent with sensory analysis of champagne and sparkling wines, since it is generally accepted that the smell of champagne, and especially its first nose, is always more irritating (because more concentrated in gaseous CO_2_ which is a strong trigeminal stimulus) when champagne is served into a flute than when it is served into a coupe. The influence of champagne temperature was also tested. As could have been expected, lowering the temperature of champagne was found to decrease ethanol vapor concentrations in the headspace of a glass. Nevertheless, and quite surprisingly, this temperature decrease had no impact on the level of gaseous CO_2_ found above the glass. Those results were discussed on the basis of a multiparameter model which describes fluxes of gaseous CO_2_ escaping the liquid phase into the form of bubbles.

## Supporting Information

Table S1 **Listing of the chromatographic parameters used for the simultaneous analysis of CO_2_ and ethanol above the free surface of champagne glasses (redrawn from** [**39**] **).**
(DOC)
