# Supplementary material for: Monitoring Gaseous CO2 and Ethanol above Champagne Glasses: Flute versus Coupe, and the Role of Temperature
Source: PLoS One. 2012 Feb 8;7(2):e30628. doi: 10.1371/journal.pone.0030628 (PMC3275598; doi:10.1371/journal.pone.0030628)
Supplement: Table S1 — Listing of the chromatographic parameters used for the simultaneous analysis of CO2 and ethanol above the free surface of champagne glasses (redrawn from [39] ). (DOC) [file pone.0030628.s001.doc]

|  | **CO_2_** | **ethanol** |
| --- | --- | --- |
| **Channel** | A | B |
| **Column** | PPU | OV-1 |
| Method acquisition parameters | | |
| **Column temperature (°C)** | 140 | 100 |
| **Sampling time (s)** | 10 | |
| **Injection time (ms)** | 50 | |
| **Detector sensibility** | low | high |
| **Running time (s)** | 60 | |
| Peak integration parameters | | |
| **Slope sensitivity (µV/s)** | 5.00 | |
| **Peak width (s)** | 0.50 | |
| **Integration OFF at time (s)** | 0.00 | 0.00 |
| **Integration ON at time (s)** | 23.50 | 30.00 |

**Table S1:** Listing of the chromatographic parameters used for the simultaneous analysis of CO_2_ and ethanol above the free surface of champagne glasses (redrawn from [39]).
